# Expanding molecular diagnostic capacity for COVID-19 in Ethiopia: operational implications, challenges and lessons learnt

**DOI:** 10.11604/pamj.2021.38.68.27501

**Published:** 2021-01-20

**Authors:** Adisu Kebede, Betty Lanyero, Berhane Beyene, Mayur Lalji Mandalia, Daniel Melese, Feven Girmachew, Adamu Mekonnen, Gonfa Ayana, Nebiyou Yemanebirhan, Getnet Hailu, Habtamu Asrat, Negash Nurahmed, Andargachew Gashu, Kirubel Eshetu, Zewdu Assefa, Aschalew Abayneh, Emmanuel Musa, Ebba Abate

**Affiliations:** 1 Ethiopian Public Health Institute, Addis Ababa, Ethiopia,; 2 World Health Organization Ethiopia Country Office, UNECA Compound, Zambezi Building, Addis Ababa, Ethiopia,; 3 World Health Organization Liaison Office to the African Union and the UN Economic Commission for Africa, UNECA Compound, Zambezi Building, Addis Ababa, Ethiopia,; 4 World Health Organization Regional Office for Africa, Brazzaville, Republic of Congo

**Keywords:** COVID-19, laboratory, reverse-transcriptase polymerase chain reaction

## Abstract

Efforts towards slowing down coronavirus (COVID-19) transmission and reducing mortality have focused on timely case detection, isolation and treatment. Availability of laboratory COVID-19 testing capacity using reverse-transcriptase polymerase chain reaction (RT-PCR) was essential for case detection. Hence, it was critical to establish and expand this capacity to test for COVID-19 in Ethiopia. To this end, using a three-phrased approach, potential public and private laboratories with RT-PCR technology were assessed, capacitated with trained human resource and equipped as required. These laboratories were verified to conduct COVID-19 testing with quality assurance checks regularly conducted. Within a 10-month period, COVID-19 testing laboratories increased from zero to 65 in all Regional States with the capacity to conduct 18,454 tests per day. The success of this rapid countrywide expansion of laboratory testing capacity for COVID-19 depended on some key operational implications: the strong laboratory coordination network within the country, the use of non-virologic laboratories, investment in capacity building, digitalization of the data for better information management and establishing quality assurance checks. A weak supply chain for laboratory reagents and consumables, differences in the brands of COVID-19 test kits, frequent breakdowns of the PCR machines and inadequate number of laboratory personnel following the adaption of a 24/7 work schedule were some of the challenges experienced during the process of laboratory expansion. Overall, we learn that multisectoral involvement of laboratories from non-health sectors, an effective supply chain system with an insight into the promotion of local production of laboratory supplies were critical during the laboratory expansion for COVID-19 testing. The consistent support from WHO and other implementing partners to Member States is needed in building the capacity of laboratories across different diagnostic capabilities in line with International Health Regulations. This will enable efficient adaptation to respond to future public health emergencies.

## Perspective

The coronavirus disease (COVID-19) is contributing to significant morbidity and mortality globally. Of the over one million deaths are reported to World Health Organization (WHO) by Member States, nearly a quarter are from the African continent [1]. In Ethiopia, over 95,000 confirmed cases and 1,400 deaths have been reported as of 29 October 2020 [2]. Efforts towards slowing down COVID-19 transmission and reducing mortality have focused on timely case detection, isolation and treatment. The availability of capacity for laboratory confirmation of COVID-19 is a cornerstone for timely case detection. WHO released interim guidance on laboratory testing for COVID-19 in suspected humans in as early as January 2020 [3]. Laboratory diagnosis using nucleic acid amplification tests (NAATs) such as reverse-transcriptase polymerase chain reaction (RT-PCR) was recommended as the preferred modality for confirming COVID-19 [3]. However, many countries including Ethiopia did not have the capacity to test for COVID-19 as shown by the readiness assessment conducted by WHO in February 2020 [4]. Furthermore, in resource limited settings, RT-PCR technology which is also resource intensive may have been limited. Therefore, countries needed to strategize and collaborate to enhance their capacities to conduct laboratory diagnosis for COVID-19 while building on existing International Health regulations (IHR) laboratory capacities. Over the years, Ethiopia has heavily invested in building laboratory capacities in line with IHR. A recent national self-assessment report [5, 6] revealed that laboratory biosafety and biosecurity guidelines and regulations were in place and implemented by all laboratories at the national level. Additionally, access to laboratory testing capacity for all priority epidemic-prone diseases were available and systems were in place for at least 80% of all health facilities to transport specimens to reference laboratories [5]. More specifically in the field of virology, a national hospital-based sentinel surveillance system for severe acute respiratory infections (SARI) / influenza like illness (ILI) was established since 2008 in eight sites in the country supported by the National Influenza, Arbovirus and Viral Hemorrhagic Fever (VHF) Reference Laboratory for confirmatory diagnosis [7]. This laboratory also conducts testing for other respiratory viruses, arboviruses, Hepatitis A and E viruses and other coronavirus strains with the exception of SARS and COVID-19. It was critical to establish COVID-19 testing capacity as one of the priority actions given the high risk of importation and spread of the SARS-CoV-2 virus in Ethiopia. Moreover, with its vast size occupying 1,127,127 km^2^ and an estimated population of 110 million inhabitants, further expansion of testing capacity at the Regional States was essential to meet the demands of testing for timely case detection. This would limit sample transportation challenges from hard-to-reach districts to the national influenza reference laboratory located in the capital city, Addis Ababa. In this article, we document the process of expansion of molecular laboratory testing capacity for COVID-19 using RT-PCR in Ethiopia. We highlight the operational implications, challenges faced, and lessons learnt. The information shared could be used to guide public health laboratory testing establishment and access expansion for future health emergences.

### The process of expansion of molecular laboratory capacity for COVID-19 testing

**Figure 1:**
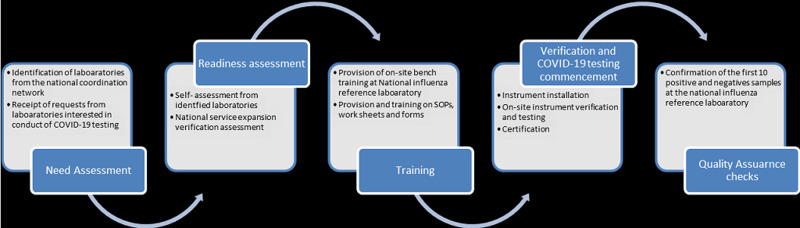
steps undertaken in the verification of laboratories to conduct COVID-19 testing in Ethiopia: potential public and private laboratories with RT-PCR technology were identified through the laboratory coordination network and requests from other laboratories interested to conduct COVID-19 testing; five major steps, namely; needs assessment, readiness assessment, training, verification and certification and quality assurance checks were undertaken to facilitate each identified laboratory to conduct COVID-19 testing; external and internal quality assurance assessments were conducted regularly; this process was led by a team of experts from EPHI, FMOH, WHO and Africa CDC

The process of laboratory expansion was led by the Federal Ministry of Health (FMOH) and Ethiopian Public Health Institute (EPHI) under the laboratory pillar in the COVID-19 incident management system. The initial step to establishing COVID-19 testing in Ethiopia was the development of a national strategy and guidance for the laboratory diagnosis of COVID-19. This was adapted from WHO guidance on laboratory investigation for COVID-19 in humans [3] and laboratory testing strategy recommendations for COVID-19 [8]. The national laboratory strategy highlighted 10 major approaches: (i) enhancement of laboratory test expansion, (ii) strengthening supply chain management system, (iii) improving specimen management system, (iv) implementing quality assurance measures, (v) strengthening laboratory equipment management, (vi) improving laboratory information and data management system, (vii) strengthening biosafety and biosecurity measures, (viii) evaluation and introduction of new test methods and technologies, (ix) monitoring, evaluation and performance improvement and (x) promoting and strengthening multi-sectoral collaboration and partnerships. The initial step of the laboratory expansion for COVD-19 testing as illustrated in Figure 1 was the identification of potential laboratories in the public and private sectors followed by assessment of their capacities. Assessments were conducted by a team of laboratory experts drawn from EPHI, regional health bureaus (RHBs) and WHO using the laboratory assessment tool [9]. The tool evaluated eight key areas: (i) organization and management of the laboratory, (ii) documentation, (iii) specimen collection, handling and transport, (iv) data and information management, (v) consumables and reagents, (vi) equipment, (vii) facility infrastructure, (viii) human resources in quality and quantity and (ix) biological risk management. Based on the assessment gap analysis, corrective actions were undertaken before testing for COVID-19 could commence. Additionally, capacity building was provided through onsite bench-training, on-site mentorship and drills for the laboratory personnel on standard operating procedures (SOPs) for COVID-19 testing. It was observed that calibration adjustments were required because laboratories had different PCR machines and differences in the brands of COVID-19 test kits donated. A verification exercise was conducted upon meeting all the requirements for setup of the laboratory for COVID-19 testing. Quality assurance was conducted using samples of known test results and the laboratory was designated as a COVID-19 testing laboratory with an official letter of approval issued to the facility by EPHI. Using a three-phased approach, Ethiopia expanded its laboratory testing capacity for COVID-19 to all 10 Regional States and two city administrations.

### Phase I

**Figure 2:**
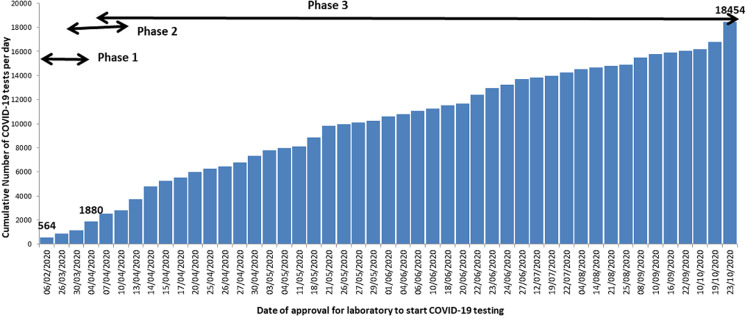
graph showing the cumulative number of COVID-19 testing capacity over a nine month period from February to October 2020: using a three phrased approach, COVID-19 testing capacity was expanded from zero to 18,454 tests conducted per day; during phase one, the National Influenza, Arbovirus and Viral Hemorrhagic fever Reference Laboratory was capacitated to conduct an average of 564 COVID-19 tests per day; phase two focused on capacitating three additional laboratories at the Ethiopian public health institute at national level with an increase of up to 1880 per day; phase three concentrated on expanding COVID-19 testing capacity in all the Regional States; this phase included a total of 61 laboratories across the country from health and non-health sectors; COVID-19 testing capacity increased from 1880 in phase two to 18,454 by the end of phase three

The first phase focused on capacitating the National Influenza, Arbovirus and VHF Reference Laboratory at EPHI to establish COVID-19 testing. This biosafety level two [2] laboratory, established since 2008 [7, 10], was selected to start COVID-19 diagnosis for the country given the availability of trained human resource in RT-PCR technology and established infrastructure. With technical guidance by WHO, steps highlighted in Figure 1 were undertaken to enable the laboratory start COVID-19 testing including the development of standard operating procedures, capacity building for the laboratory professionals, evaluation of the first COVID-19 test kits supplied by WHO and calibrations of the PCR machines. Additional guidance documents on COVID-19 laboratory quality assurance, protocols for specimen pooling and management and a results communication protocol were developed. On 6^th^ February 2020, Ethiopia conducted its first COVID-19 testing at the National Influenza, Arbovirus and VHF Reference Laboratory. Phase one of expansion of COVID-19 laboratory capacity resulted into the establishment of the first testing laboratory in the country with an average of 564 tests per day (Figure 2).

### Phase II

Phase II focused on laboratories at the national level with capacity to conduct molecular testing such as the national HIV, bacteriology and parasitology molecular laboratories. Learning from establishment of the National Influenza, Arbovirus and VHF Reference Laboratory, gaps identified during the assessments were addressed and on-site bench training conducted for laboratory experts from the three laboratories. Within a three-month period, the number of laboratories testing for COVID-19 increased from one to four at the national level with an increase from an average of 564 to 1,880 tests per day (Figure 2).

### Phase III

**Figure 3:**
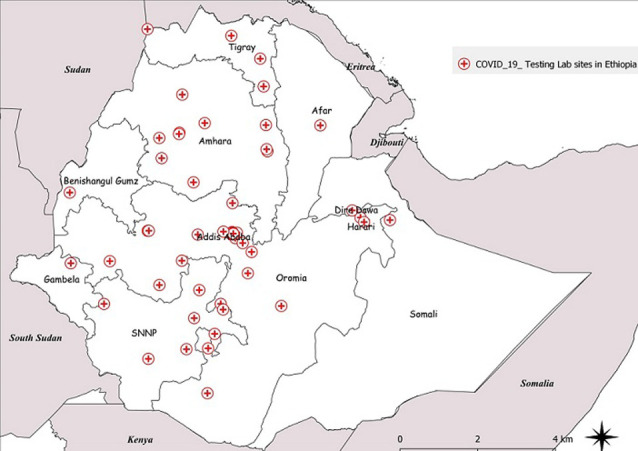
distribution of COVID-19 testing laboratories in Ethiopia; COVID-19 molecular testing capacity using RT-PCR was expanded from zero to 65 laboratories distributed across all 10 regions and two city administrations; these included laboratories with RT-PCR technology from public health and research institutions and hospitals under the FMOH, universities and research institutions under the Ministry of Science and Higher Education, Ministry of Agriculture, Ministry of Defense and private health facilities; Data source: Ethiopian Public Health Institute

Phase III focused on expansion of COVID-19 testing to the regional level laboratories in the 10 Regional States and two city administrations. There were a total of 13 public health laboratories under the FMOH and the respective RHBs for public health emergencies prior to the laboratory expansion. Using the steps highlighted in Figure 1, laboratories were capacitated to start COVID-19 testing. Priority for expansion for this phase was started in Addis Ababa because of the high demand for testing. During this time, March and April 2020, more than 70% of the COVID-19 cases during were reported from Addis Ababa. Subsequently, laboratory expansion commenced to all the Regional States with experts at the national level, WHO and implementing partners supporting the process. In addition to the public health laboratories under the FMOH, phase III included expansion of COVID-19 testing to laboratories with RT-PCR capacity belonging to research institutions and universities under the Ministry of Agriculture and Ministry of Science and Higher Education. Moreover, other laboratories included those from major hospitals such as Black Lion (Tikur Anbessa) and ALERT hospitals in Addis Ababa and private laboratories such as International Clinical Laboratories, Silk Road Hospital Laboratory, PANVAC laboratory owned by the African Union. Of the total 65 laboratories conducting COVID-19 testing, 24 (37%) are public health and research institution laboratories and hospital-based laboratories under the FMOH, 28 (43%) are from universities and under the Ministry of Science and Higher Education, 4 (6%) under the Ministry of Agriculture, 1 (2%) under the Ministry of Defense and 8 (12%) from private facilities. By October 2020, the testing capacity had expanded to 18,454 tests per day from 65 COVID-19 testing laboratories throughout the country as shown in Figure 3.

### Operational implications

The rapid country-wide expansion of COVID-19 molecular laboratory testing posed some operational implications given that this was the first time such an intervention was embarked upon in the country during a health emergency situation. Firstly, is the organization of the coordination of all laboratories for the response. Before the pandemic, the laboratory coordination network established by the National Laboratories Capacity Building Directorate under EPHI administered the work done by the national and regional public health laboratories. During the expansion process, laboratories from non-health sectors were included in this coordination network to strengthen and streamline the COVID-19 testing work. Additionally, the advocacy and directive from the Prime Minister´s level Task Force to engage different sectors in the expansion of the laboratory reinforced efforts for a coordinated approach. Secondly, was the use of non-virologic molecular laboratories. Prior to the COVID-19 pandemic, FMOH, EPHI and WHO had invested heavily in capacity building on molecular technology for the national referral laboratories such as national polio laboratory, measles and rubella laboratory, rotavirus laboratory, pediatric bacterial meningitis laboratory and National Influenza, Arbovirus and VHF Reference Laboratory. Utilization of these laboratories not only provided COVID-19 testing but saved on resources that may have otherwise been needed to be mobilized to procure machines, setup of additional laboratories or hire trained laboratory professionals. In addition to conducting COVID-19 testing, the laboratories also maintained their critical functions offering testing for HIV, tuberculosis, vaccine derived polio virus, meningitis, and malaria among others. Thirdly, the investment in capacity building has resulted in long term benefits. Significant investments and efforts were made in enhancing the knowledge and skills of laboratory professionals to conduct COVID-19 testing. With support from WHO and the African Centres for Disease Control and Prevention (Africa CDC), one laboratory expert was trained in Senegal in January 2020 together with other experts from different African countries. Later in February 2020, an international laboratory expert conducted national level training-of-trainers´ course for laboratory professionals from EPHI with support from WHO. From then on, EPHI in collaboration with WHO and Africa CDC facilitated the capacity building of four laboratory professional for COVID-19 testing. Capacity building provided through classroom based training, on-site bench training, on-site mentorship, drills and webinars contributed to strengthening the knowledge and skills of laboratory professionals. Furthermore, capacity building was provided for public health emergency management (PHEM) surveillance officers, rapid response team members, hospital staff and various cadres of personnel on sample collection triple packaging based on IATA Dangerous Goods Regulation, biosafety and waste management. Over 500 laboratory and health professionals were trained to support the sample collection in communities, health facilities, quarantine facilities, isolation facilities where suspect cases where found. Fourthly, was the institutionalization and digitalization of the data and information management. The paper-based information flow from sample reception till result delivery was challenging and increased the risk of contamination for the health workers. The FMOH introduced laboratory data indicators into the District Health Information System 2 (DHIS 2) to facilitate digitalization of COVID-19 laboratory related data [11]. DHIS 2 is a tool for collection, validation, analysis, and presentation of aggregate and patient based statistical data, tailored to integrated health information management activities [12]. Use of the DHIS 2 tool enabled real time data review, analysis and utilization at points of collection and at various administrative levels for decision making. It also contributed to improved turnaround time for the COVID-19 test results. Lastly, maintaining quality in all the expanded laboratories for the COVID-19 testing. External Quality Assessment (EQA) comprising of proficiency testing was conducted for samples from the National Influenza, Arbovirus and VHF Reference Laboratory at the National Institute for Communicable Diseases for South Africa reference laboratory (NICD). NICD is one of WHO´s regional reference laboratories. The National Influenza, Arbovirus and VHF Reference Laboratory scored 100% on the EQA conducted in April 2020 and September 2020. Internally, proficiency testing panels arranged from the pooled positive and negative samples were prepared by the National Influenza, Arbovirus and VHF Reference Laboratory. Using this panel, over 30 laboratories conducting COVID-19 testing scored 100% while a few scored above 80%. Overall, the quality of COVID-19 testing was maintained above the required standards. Moreover, regular supportive supervision visits were conducted by a team of laboratory experts from EPHI and WHO.

### Challenges

During the process of laboratory testing expansion for COVID-19 some key challenges were faced. A weak supply chain management for laboratory reagents and supplies was a major concern. As a result, there were interruptions in COVID-19 testing, back logs of untested samples and prolonged waiting time for sample collection from suspect cases. These delays could have increased the risk of spread of COVID-19 infection as suspect cases were not timely tested. Additionally, disruption of the global supply chain mechanism during the pandemic contributed to delays in procurement of laboratory supplies and consumables. Differences in the brand of the COVID-19 test kits necessitating frequent configuration of the molecular testing machines was another challenge. The difference in the brands of COVID-19 test kits required that the PCR machines are re-configured and laboratory personnel orientated on the new protocols. This increased the likelihood of error and a longer turnaround time for results because additional time was spent on re-configurations and orientations. Frequent breakdowns of the PCR testing machines were also a challenge. Due to the increased demands for testing, the PCR machines were run 24/7 resulting in machine breakdown and the need for frequent maintenance. Additionally, technicians for the PCR machines and spare-parts that were not readily available in-country had to be sourced out of the country. This contributed to further delays in testing. As an interim measure, pending samples for testing were transported to the nearest laboratory for processing. This highlighted the need to strengthened capacity for preventive maintenance and corrective maintenance at national level to minimize interruptions. Lastly, the inadequate number of laboratory personnel with expertise in molecular technology to meet the demands of the workload for the expanded COVID-19 testing laboratories. A 24/7 work schedule for laboratory personnel was adapted to meet the testing demands, however, this overstretched the limited number of trained personnel. The laboratory professionals were fatigued which could compromise the quality of work. Thus, to ensure quality is maintained there is need to build capacity of a critical mass of laboratory personnel on molecular technology and consider provision of incentives for the laboratory professionals who work under high-risk environment and long hours during emergencies.

### Lessons learnt

We learnt that the multisectoral involvement and commitment significantly contributed to the achievements in the expansion of laboratory diagnosis for COVID-19 in Ethiopia. The laboratories and technical experts from the health and non-health sectors provided the needed resources to support the laboratory expansion for COVID-19 testing. Secondly, establishment of an effective supply chain management system for health emergency supplies including laboratory reagents is vital during response. Reiterating that laboratory testing is a cornerstone for timely case detection in a disease outbreak, maintaining consistent supply of laboratory reagents and supplies and all aspects in the supply chain management is critical. For example, in Ethiopia, strengthening of a courier system could support transportation of samples and supplies from the health facility, woreda level, and regional level to the national level and vice versa thus limiting the risk of infection during transportation of infectious samples and timely delivery of samples to the laboratories. Thirdly, is the importance of mapping and regularly updating of laboratory resources and technical expertise available in the country from the public and private sectors. Availability of this database enables the country to know the resource gaps for which appropriate measures can be undertaken as part of preparedness for health emergencies. As noted during the laboratory expansion, some of the laboratories have only one machine. It is important to prioritize strengthening of laboratory capacities in these regions. Lastly, is the need to explore and promote local production for PCR reagents and laboratory supplies. Only as of September 2020, several months after the COVID-19 pandemic was reported in the country, had the country established a plant that could manufacture COVID-19 test kits. Efforts to promote local production of PCR reagents and laboratory supplies could significantly reduce the supply shortages.

## Conclusion

Expanding molecular laboratory testing capacity for COVID-19 in Ethiopia from zero to 65 laboratories as of October 2020 conducting up to 18,454 tests per day contributed to improved case detection for COVID-19 in the country. The use of a well-coordinated, systematic and phased approach with multisectoral collaboration and partnerships as well as effective technical, operational and logistic support facilitated the expansion. Consistent support from WHO and other implementing partners to Member States in strengthening laboratory capacities across different diagnostic disciplines in line with International Health Regulation will enable efficient adaptation to future public health emergencies.
